# Nutritional stress exacerbates impact of a novel insecticide on solitary bees' behaviour, reproduction and survival

**DOI:** 10.1098/rspb.2022.1013

**Published:** 2022-10-12

**Authors:** Anina C. Knauer, Cedric Alaux, Matthew J. Allan, Robin R. Dean, Virginie Dievart, Gaétan Glauser, Tomasz Kiljanek, Denis Michez, Janine M. Schwarz, Giovanni Tamburini, Dimitry Wintermantel, Alexandra-Maria Klein, Matthias Albrecht

**Affiliations:** ^1^ Agroecology and Environment, Agroscope, Zürich, Switzerland; ^2^ UR406 Abeilles and Environnement, Site Agroparc, INRAE, Avignon, France; ^3^ Atlantic Pollination Ltd, Eastleigh, UK; ^4^ Red Beehive Company, Bishops Waltham, UK; ^5^ Neuchâtel Platform of Analytical Chemistry, University of Neuchâtel, Neuchâtel, Switzerland; ^6^ Department of Pharmacology and Toxicology, National Veterinary Research Institute, Pulawy, Poland; ^7^ Institute for Biosciences, University of Mons, Mons, Belgium; ^8^ Department of Soil, Plant and Food Sciences (DiSSPA—Entomology), University of Bari, Bari, Italy; ^9^ Nature Conservation and Landscape Ecology, University of Freiburg, Freiburg, Germany

**Keywords:** bee health, foraging, nectar, pesticide, pollen, reproduction

## Abstract

Pesticide exposure and food stress are major threats to bees, but their potential synergistic impacts under field-realistic conditions remain poorly understood and are not considered in current pesticide risk assessments. We conducted a semi-field experiment to examine the single and interactive effects of the novel insecticide flupyradifurone (FPF) and nutritional stress on fitness proxies in the solitary bee *Osmia bicornis*. Individually marked bees were released into flight cages with monocultures of buckwheat, wild mustard or purple tansy, which were assigned to an insecticide treatment (FPF or control) in a crossed design. Nutritional stress, which was high in bees foraging on buckwheat, intermediate on wild mustard and low on purple tansy, modulated the impact of insecticide exposure. Within the first day after application of FPF, mortality of bees feeding on buckwheat was 29 times higher compared with control treatments, while mortality of FPF exposed and control bees was similar in the other two plant species. Moreover, we found negative synergistic impacts of FPF and nutritional stress on offspring production, flight activity, flight duration and flower visitation frequency. These results reveal that environmental policies and risk assessment schemes that ignore interactions among anthropogenic stressors will fail to adequately protect bees and the pollination services they provide.

## 1. Introduction

Declines of wild bees are threatening the pollination services they provide to entomophilous plant species, including over 75% of the most important global crops [1,2]. A widespread use of pesticides and loss of floral food resources accompanying agricultural intensification are considered major drivers of these declines [1,3,4]. Pesticides, especially insecticides, can have manifold lethal and sublethal effects on bees, such as impaired reproduction, orientation or memory [5,6]. Similarly, limited flower availability and diversity can reduce survival and reproduction of bees [7–11]. As a result, these drivers may even reinforce each other through synergistic negative impacts on bees [12,13]. Understanding how interactions between pesticides and food stress impact bees in the field is one of the main challenges in developing effective measures to mitigate bee declines.

Laboratory studies have provided some evidence that poor nutrition and pesticide exposure can synergistically reduce bee survival [12]. For example, honeybees with limited access to carbohydrates or pollen showed an increased susceptibility towards insecticides [14–17], while bees that fed on a diet with a low protein-lipid ratio were more resilient to pesticide stress [18]. Some secondary metabolites in the pollen can further induce the detoxification system in bees, which helps them to eliminate pesticides and better withstand their detrimental effects [19–21]. Also, pesticides can alter nutritional physiology and affect food consumption rates, foraging success and flower preferences, which may reinforce nutritional stress and contribute to a synergism between these stressors [22–25]. However, studies examining interactive effects of pesticide exposure and nutritional stress on bees under field-realistic conditions are largely lacking [8,25].

The current risk assessment process by which novel pesticides get licensed only considers bee toxicity (LD_50_) of individual agrochemicals and, if higher tier (semi-)field studies are carried out, their negative effects on proxies of fitness such as colony development and survival of social bees [26,27]. Potential amplifications by other stressors to which bees are frequently co-exposed in intensively managed agroecosystems (e.g. food stress) are not considered [28,29]. Risk assessment furthermore relies on a few model species, mostly the honeybee *Apis mellifera*, to assess toxicity for pollinators. Yet, honeybee LD_50_ values may not be representative for all pollinator species [29] as life-history traits can modulate species' sensitivity and exposure to different agrochemicals with consequences for population development [30,31]. While social bee species may compensate for temporary negative effects of agrochemicals at the colony level at a later point in time [32], negative effects should directly impair the reproductive output in solitary bee species [31]. Therefore, attempts are currently made by environmental policies to establish solitary bees from the genus *Osmia,* which contains important crop pollinators, as model species for pesticide risk assessment [26,33].

To test the hypothesis that food stress can augment the impact of an insecticide on the performance of the solitary bee *Osmia bicornis* in field-realistic conditions, we conducted a semi-field experiment supplemented with laboratory measurements. Bees were released into large flight cages of 54 m^2^ supplied with flowering monocultures of buckwheat (*Fagopyrum esculentum*), wild mustard (*Sinapis arvensis*) or purple tansy (*Phacelia tanacetifolia*) (electronic supplementary material, figure S1*a*–*c*). These plant species frequently occur in agricultural landscapes and represent suitable candidates for (semi-)field risk assessment studies with *Osmia* model species because they can be sown in spring and have an early onset of flowering that overlaps with the bees' reproductive period. Per plant species, half of the flight cages were sprayed with FPF (product Sivanto Prime, Bayer Crop Science) according to label guidelines [34] in a crossed factorial design. FPF is a novel systemic insecticide that has been registered for use against sucking pests in a wide variety of crops including bee-attractive fruit trees, legumes and vegetables. It can be applied via spray application, drench or seed treatment and is used in broad geographical regions globally including countries in North and South America, Asia, Africa, Europe and Australia. It belongs to the chemical class of butenolides, but the mode of action is comparable to that of neonicotinoids, targeting nicotinic acetylcholine receptors, which makes FPF a potential successor of the partially banned neonicotinoids [35]. As FPF has been labelled as bee-safe, it can be sprayed into flowering bee-attractive crops [34,36,37]. Currently, (semi-)field studies on the consequences of FPF exposure on solitary bees are lacking. Such studies are, however, urgently needed, considering the lower LD_50_ values of FPF for solitary bee species compared to honeybees [38,39].

## 2. Material and methods

### (a) Study organism

Various species in the genus *Osmia* are important crop pollinators [40]. The red mason bee *Osmia bicornis* is a common European solitary and univoltine bee species nesting in pre-existing cavities. Brood cells are provisioned with a mixture of pollen and nectar to feed the developing offspring. Pollen from 19 plant families have been identified in their nests [41], but usually a single brood cell contains only few different pollen taxa [42]. After the consumption of the pollen-nectar provision, larvae spin a cocoon and pupate. The bees overwinter as adults inside the cocoon and start emerging in spring. Bees for this experiment were collected as cocoons from several local sites in Switzerland (obtained from Wildbiene + Partner AG, Switzerland).

### (b) Experimental design

The experiment was conducted with a total of 432 individually marked *Osmia bicornis* females (see below) in 9 × 6 m flight cages (height: 2 m; steel frame covered with transparent nylon netting of *ca* 1.15 mm mesh size; Howitec Netting, The Netherlands) at an experimental field site near Zürich (Switzerland) in mid-June 2020. We established three food plant species: buckwheat (*Fagopyrum esculentum*), wild mustard (*Sinapis arvensis*) and purple tansy (*Phacelia tanacetifolia*) (electronic supplementary material, figure S1)*.* Each of these nutrition treatments was assessed in combination with two insecticide treatments: spray application of the product Sivanto Prime (Bayer Crop Science, containing 200 g l^−1^ of FPF) versus control treatment (no FPF applied). Three cages were assigned to each treatment combination in a crossed design for a total of 18 cages. Cages were distributed with 5 m distances between cages and from field boundaries. To control for any soil or light gradient in the field, each plant species was represented once in each column and row of a 6 × 6 array block and insecticide treatments (FPF application or control) alternated between columns and rows (electronic supplementary material, figure S1*d*). Plants were sown at rates recommended for arable fields (buckwheat: 200 g a^−1^, wild mustard: 100 g a^−1^, purple tansy: 60 g a^−1^) without standardizing the plant species' flower abundance, which was considered to be one property affecting nutritional stress (see below for quantification). In each cage, a nesting unit with 120 cavities was installed at 1.2 m height. Nesting units were composed of 12 layers (1.8 cm MDF boards) with 10 cavities (8 × 8 mm) each (electronic supplementary material, figure S1*f*). Cavities drilled into the MDF boards were half-round and open on top to allow observation of nesting progress. To ensure that bees and nests were not disturbed during observations, each layer was covered with a transparent plastic foil (electronic supplementary material, figure S1*f*). All nesting units were placed on the opposite side of the cage entrance, oriented southeasterly and shaded with a wooden roof. Besides each nesting unit a hole was dug into the ground, which was regularly filled with water and offered the females mud for nest construction. Three additional cages were used to cultivate all three food plants together (not treated with FPF), in which food plant foraging preferences were measured. Each plant species covered the same area (parallel strips of 7.5 × 1.5 m with identical minimum distance of 2 m of nearest plants to the nesting unit) and was sown with the same rates as used for monocultures. Moreover, one additional cage of each monocultural food plant (treated with FPF) was used for destructive measurements, such as sampling pollen-nectar provisions from nests and bees, for example, pesticide residue analysis.

Three weeks after FPF application, the nesting units were covered with a fine mesh (0.5 mm × 0.5 mm) to prevent parasitism or predator attack and carefully transported from the field site to the storage place (outdoor, shaded and protected from rain and heat) where the offspring hibernated. In February 2021, nests were transferred to a cold chamber (4°C) and the offspring was hatched in April at room temperature.

### (c) Bee rearing

All plant species started flowering in late May within three subsequent days. At the beginning of flowering, the rearing of the *O. bicornis* cocoons was commenced. Bees were incubated at room temperature in small hatching cages (60 × 60 × 60 cm; BugDorm, USA), separated by sex (separation was based on cocoon diameter: male < 6 mm and female ≥ 6 mm). As males emerge faster, the incubation of males was started 3 days after the incubation of females. The hatching cages were checked daily and emerged bees were transferred to a cold chamber (4°C) where they remained until release. All bees that hatched on the same day were randomly distributed to the different cages in equal proportions to avoid any bias of cold storage on treatments. Females were marked with a digit from 1 to 8 in three different colours (yellow, white, green; marking kit for honeybee queens, Imkereibedarf Wespi GmbH, Switzerland) (electronic supplementary material, figure S1*e*). Each colour-digit combination was represented once per cage and allowed individual recognition of nesting females and thus per female assessments of survival and fitness proxies. Four days after incubation had been started, a total of 24 females and 36 males were released per cage resulting in a sample size of 72 females per treatment. To ensure simultaneous initiation of nesting, bees in all cages were released on the same day. This coincided with a sufficient floral food supply in all cages (roughly 15% of plants having open flowers). Five days after the release, female *O. bicornis* had started nesting in all the cages. In the additional cages with the mix of all three food plants 24 individually marked females and 36 males were released also, while in each of the three additional cages with monocultures for destructive measurements, 100 unmarked females and 80 males were released.

### (d) Insecticide application

Insecticide application was done about one week after bees were released into cages. Following guidelines by the International Commission for Plant–Pollinator Relationships (ICPPR) non-*Apis* working group [33] for semi-field risk assessments using *Osmia* spp., insecticide application was done after the majority of female *O. bicornis* had started nesting in all the cages (at least 16 nests were initiated in all cages), which is necessary to properly study the impact of pesticide exposure on reproduction. FPF (Sivanto Prime, Bayer Crop Science) was applied at the highest recommended rate of 205 g active ingredient per ha according to the product label guidelines in the early morning before full bee flight [34]. The percentage of foraging females during application was estimated to be below 10% compared to full bee flight (i.e. a maximum of three foraging females were observed per cage). The spray application was done by a certified ecotoxicological risk assessment company (Innovative Environmental Services (IES) Ltd.) in dry weather and with wind speed lower than 3.0 m s^−1^. To ensure an even application of the product, spraying was performed using a motorized backpack sprayer equipped with anti-drift spraying nozzles. Nesting units were covered with plastic foil during spraying to prevent spray drift to *O. bicornis* nests. The exact volume of product applied was measured and recorded after application of the product in each cage. An equal volume of water was sprayed to control cages before insecticide application to avoid any contamination. Plant surfaces dried in less than 30 min in all treatments and no differences between plant species could be observed.

### (e) Plant properties and nutritional stress

Food plants were selected along a gradient of food quantity and quality relevant for bees [e.g. 43–45]. To confirm these properties and characterize nutritional quantity and quality for all plant species, we measured flower abundance (for each assessment day), nectar volume and sugar content, pollen amount and pollen phenolic compounds, glucosinolates and protein and lipid content (see electronic supplementary material for detailed description of the method).

As the relative importance of these different properties in driving nutritional stress for *O. bicornis* bees remains largely unknown until now, we used measurement of bee health related to nutrition that can be taken from bees directly. The following three measurements were selected to assess nutritional values of buckwheat, wild mustard and purple tansy: (i) the bees' preference to forage on each food plant when all three species were available (measured in cages with strips of all three plant species) [46,47], (ii) the gene expression of vitellogenin in bees foraging on monocultures of the three food plants (measured in the additional monocultural cages) [10,17,48] and (iii) the bees' ability to clear FPF after foraging on monocultures of the three food plants (measured in the additional monocultural cages) [19,49]. A detailed description of how these measurements were obtained can be found in the electronic supplementary material. As all three measures positively relate to the health and nutritional status of bees, nutritional value of food plants was determined as the first principal component (capturing 82% of variability) from a principal component analysis (PCA) on these measures. To obtain a measure of nutritional stress instead of nutritional value, the axis of the obtained first principal component was reversed (negative values were turned into positive and *vice versa*).

### (f) *Osmia bicornis* proxies of fitness

All per-female proxies of fitness of *O. bicornis* were assessed during three days: the day at which FPF was applied in the morning (day 1), the subsequent day (day 2) and the ninth day after application (day 9). These days represent typical time periods to assess acute and chronic pesticide effects on bees [26]. Survival and offspring production of individual females were measured by photographing the layers of each nesting unit at night and counting the total number of roosting females [33] and the number of brood cells produced per nest cavity (electronic supplementary material, figure S1*f*). Photographs were taken before and after each day for which fitness was assessed. The nesting progress of each brood cell was rated as follows: 33%: less than half of pollen store deposited; 66%: complete pollen store deposited; 100%: egg laid and cell wall finished. Survival and nesting progress per nest were calculated as differences between values from consecutive photographs. In cages where the number of nests with nesting progress was lower than the number of alive females, we set offspring production of the remaining females to zero. The number of brood cells constructed in individual nests was used as a measure of offspring production per female. This measurement is representative as only 0.2% of nests were shared by more than one female (measured by Bee Tracker software as described below).

Flower visitation frequency was assessed for five foraging females per cage and assessment round, except when less than five females were foraging (73, 82 and 91 bees were observed during days 1, 2 and 9, respectively). Females were observed during two minutes and the number of visited flowers was recorded. When the bee was visually lost or returned to the nest after less than 1 min, the measurement was repeated. The daytime, the precise duration of the observation and the bee ID were recorded. The observations were done by three observers, which visited one cage per treatment during each assessment day to avoid any observer bias. Furthermore, software analysis of video recordings was used to analyse flight activity, flight duration and nest recognition for individual females. The front side of each nesting unit with its cavity entrances, as well as the nesting females, were filmed using high-resolution (4 K) video cameras (Legria HF G50 4 K camcorder, Canon) during several hours per assessment day (day 1: *ca* 2.5 h; day 2: *ca* 4 h; day 9: *ca* 3.5 h) which covered the peak of foraging activity (between 10.00 and 15.00). Each nesting unit was filmed simultaneously with separate cameras in each cage. Cameras were positioned at a distance of 1 m at a height of 1.5 m in front of the nesting unit using on a tripod. The produced videos were analysed with the novel machine-learning based software Bee Tracker [50]. The software is able to identify individual bee IDs and their nests (the cavity ID a female bee is nesting in). The software analysis was checked visually in the visualization videos generated by the software at four different running times during 30 s each. No errors in the output data after error correction by the software could be found. Additionally, software precision (proportion of correctly identified bees, nests and events such as leaving or entering the nest) was assessed as described in Knauer *et al.* [50] and reached 96%. Females were considered as active when leaving and returning to the nest at least once during the recording time. Flight duration represents the time from leaving the nest until a bee's return to nest (2612, 2886 and 1134 flights were recorded during days 1, 2 and 9, respectively), while nest recognition was measured as the number of cavities probed until a bee has found its own ‘correct' nesting cavity (3675, 4401 and 1967 returns to nest were recorded during days 1, 2 and 9, respectively).

For each offspring produced during the three assessment days, survival was recorded as bees that hatched in the next spring. Furthermore, the sex and weight of bees that reached adult stage (including emerged and not emerged ones) were measured.

### (g) FPF residues in pollen-nectar provisions

To determine residue levels of FPF in pollen-nectar provisions collected by *O. bicornis*, approximately 10 provisions per nutrition treatment were collected from each additional cage the night after FPF application. To ensure that only provisions collected by female bees during the same day FPF had been applied were sampled, all pollen from uncompleted brood cells was removed early in the morning before FPF application, and brood cell construction was recorded by marking newly completed brood cells on the transparent foil covering each nest layer. All samples from the same nutrition treatment were pooled for chemical analysis, which was done as described in Kiljanek *et al.* [51] (see electronic supplementary material for detailed description of the method).

### (h) Statistical analysis

To test for differences in vitellogenin gene expression levels between nutrition treatments, an ANOVA with post-hoc Dunn-Bonferroni tests was done. The preference of bees to forage on the different food plants when given a choice was analysed with a Kruskall–Wallis test with a post-hoc pairwise Wilkoxon test with Bonferroni adjustment. Differences in FPF clearance could not be analysed statistically because of the necessary pooling of samples.

To test for interactive effects of nutritional stress (see above for quantification) and the insecticide FPF on various proxies of fitness, we fitted models with the nutritional stress, the insecticide treatment (FPF application or control) and their interaction as explanatory variables. Estimates of main effects were obtained from models without interaction terms. Separate models were run to analyze data from different assessment days to test for short-term (days 1 and 2) and long-term effects after chronic exposure (day 9). Data on offspring survival, sex ratio and body weight were pooled for days 1 and 2 to obtain sufficient sample size as foraging activity and offspring production drastically dropped during these days in FPF-treated buckwheat cages.

For adult female survival, we used a generalized linear mixed-effects model (GLMM) with survival as a binary response (dead or alive) and the cage ID as random term. The same binomial GLMM structure was used to analyse flight activity (inactive or active), offspring survival (dead or hatched) and offspring sex (male or female). Nest recognition was used to distinguish between bees that immediately find their nest and bees that first have to probe other cavities and search for it. Nest recognition was therefore also analysed with a GLMM with a binomial distribution (search or find), but we additionally included time of day as a covariate and bee ID (nested in cage ID) as a random term to account for repeated measurements per female. Flight duration (log-transformed to achieve normal distribution of residuals) was analysed with a linear mixed-effects model (LMM) using the same explanatory variables and random structure as in the model for nest searching. Flower visitation frequency (square root transformed to achieve normal distribution of residuals) was also analysed with an LMM, but with observer as covariate while time of day was excluded from the model based on a likelihood ratio test (LRT). The cage ID was included as random term; bee ID was not considered as we did not collect repeated measurements per female. Finally, the number of produced offspring per nesting female (log-transformed to achieve normal distribution of residuals) and offspring weight were both analysed with an LMM including cage ID as random term. For offspring weight, we additionally included sex as covariate.

To fit models, we used the ‘lme4' [52] and ‘nlme' [53] packages implemented in R. Normality and homoscedasticity of the model residuals were validated graphically [54] and the varPower fuction was used to approach homoscedasticity where necessary. Likelihood-ratio tests were used for statistical inference [55]. To confirm the robustness of the analysis, the same models as described above but with the nutrition treatment as categorical explanatory variable (instead of nutritional stress) were fitted and revealed similar results (electronic supplementary material, tables S1–S4). Where significant interactions between FPF exposure and nutritional stress were found, Tukey post-hoc comparisons were done with the ‘emmeans' package to test for an effect of PFF exposure within nutrition treatments. All statistical analyses were done in R 4.1.0 [56].

## 3. Results

### (a) Nutritional stress

**Figure 1.**
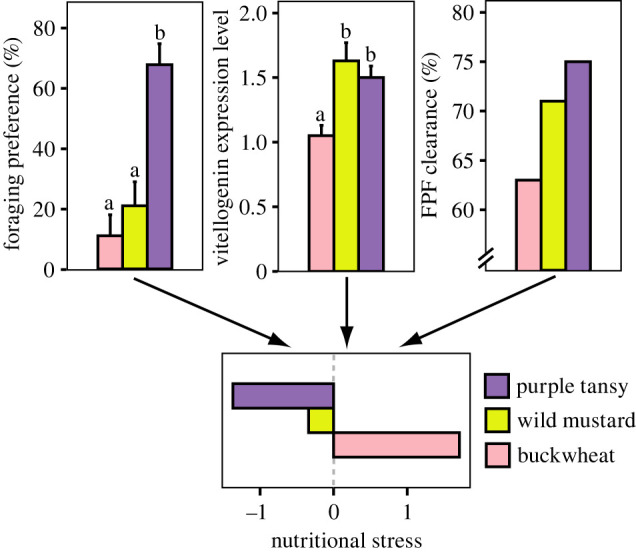
Mean values (+ s.e.) of the measurements used to assess nutritional stress for *Osmia bicornis* bees when foraging on the different food plants. Significant differences in foraging preferences and the gene expression levels of vitellogenin are indicated with different letters. (Online version in colour.)

Bees experienced the highest nutritional stress when foraging on buckwheat, while nutritional stress was intermediate on wild mustard and low on purple tansy, as determined by the first principal component of a PCA integrating (i) the bees' foraging preference when given a choice between all three food plants in additional cages, (ii) the food plant-dependent ability of bees to clear FPF after single exposure in the laboratory and (iii) the food-dependent gene expression level of vitellogenin (figure 1). Additionally, food plants also differed substantially in floral rewards and nutritional properties in nectar and pollen (table 1; electronic supplementary material, table S5). While flower abundance, nectar rewards (volume per flower and sugar content) and pollen protein contents were highest in purple tansy, this species showed intermediate pollen volumes and contents of lipids and phenolics in pollen. Wild mustard displayed intermediate values for flower abundance and nectar sugar content, but it had highest values of pollen volumes per flower and pollen lipid contents, while nectar volumes and contents of pollen proteins and phenolics were lowest compared to the other two plant species. Also, glucosinolates were only found in the pollen of wild mustard. Buckwheat had lowest values for most nutritional properties, only nectar volumes and pollen protein contents showed intermediate values and pollen phenolic contents were even highest in this species.

**Table 1.** Nutritional properties of buckwheat, wild mustard and purple tansy. GLS: glucosinolate. Amounts of single phenolic and glucosinolate compounds are provided in electronic supplementary material, table S5.

	buckwheat	wild mustard	purple tansy
**flower abundance**			
day 1	430 ± 40	1584 ± 117	1850 ± 299
day 2	433 ± 58	1524 ± 145	1791 ± 107
day 9	353 ± 33	809 ± 65	1042 ± 74
**nectar**			
volume per flower (µl)	127 ± 7	106 ± 9	197 ± 17
sugar content (%)	47	51	53
**pollen**			
volume per flower (µl)	20 ± 3	580 ± 59	540 ± 47
protein content (mg g^−1^)	161 ± 9	142 ± 7	301 ± 8
lipid content (mg g^−1^)	78 ± 5	95 ± 5	84 ± 7
protein : lipid ratio	2.1 ± 0.2	1.5 ± 0.1	3.6 ± 0.3
phenolic content (mg g^−1^)	6.6	2.5	5.3
GLS content (mg g^−1^)	0	0.87	0

### (b) Impact of nutritional stress and FPF on bees

**Figure 2.**
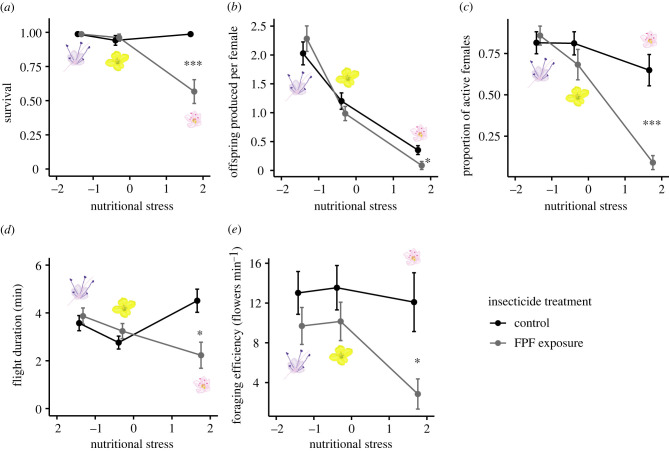
Synergistic interactions between FPF exposure (product Sivanto Prime) and nutritional stress on *Osmia bicornis.* During day 1 after FPF (and water control) application, FPF and nutritional stress synergistically impacted: (*a*) Adult female survival. (*b*) Number of offspring (brood cells) a female produced. (*c*) Proportion of active females (flight activity). (*d*) Flight duration. (*e*) Flower visitation frequency. Bars represent estimated marginal means (± s.e.). Asterisks indicate significant differences between insecticide treatments (FPF application or control) within plant species (*p < 0.05; ***p < 0.001). From left to right: purple tansy (*Phacelia tanacetifolia*), wild mustard (*Sinapis arvensis*) and buckwheat (*Fagopyrum esculentum*). (Online version in colour.)

**Figure 3.**
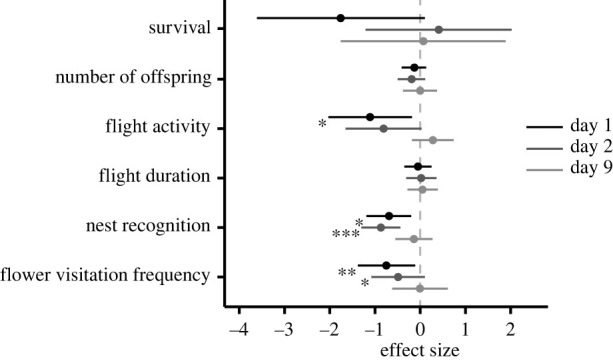
Standardized effect size of FPF exposure (product Sivanto Prime) across all three food plants on *Osmia bicornis.* Estimates are reported separately for the different assessment periods after FPF (and water control) application. Error bars represent 95% confidence intervals. Asterisks indicate significant effects (*p < 0.05; **p < 0.01; ***p < 0.001).

The effect of FPF exposure on the survival of female bees was augmented by nutritional stress on the day of pesticide application (day 1) (*λ*_LR_ = 6.57, *p* = 0.010). During this day, survival of adult female *O. bicornis* foraging on FPF-treated buckwheat was decreased by 43% compared to bees foraging on buckwheat not sprayed with FPF. In the other two plant species representing lower nutritional stress, mortality was not affected by FPF exposure despite the higher FPF residue levels (figure 2*a*). Similarly, there was a synergistic negative effect of FPF exposure and nutritional stress on offspring production per female on day 1 (*λ*_LR_ = 7.28, *p* = 0.007) and day 2 (*λ*_LR_ = 4.15, *p* = 0.042). In buckwheat cages, offspring production was reduced by 76% and 67% on day 1 and day 2, respectively, after FPF exposure, while no effect was detected in the other two food plant species (figure 2*b*). Furthermore, FPF exposure and nutritional stress synergistically negatively affected the flight activity (active classified as bees entering/leaving nest at least once during observation time) of *O. bicornis* on day 1 (*λ*_LR_ = 10.40, *p* = 0.001) and day 2 (*λ*_LR_ = 8.00, *p* = 0.005). In FPF-treated buckwheat cages, the number of active females dropped by 86% and 65% on day 1 and day 2, respectively, compared to control cages, while FPF did not affect flight activity in the other two food plants (figure 2*c*). Moreover, there was a synergistic negative effect of FPF exposure and nutritional stress on flight duration (*λ*_LR_ = 7.01, *p* = 0.008) and on flower visitation frequency (*λ*_LR_ = 4.38, *p* = 0.036) on day 1. Flight duration was decreased by 51% and flower visitation frequency by 83% in FPF-treated buckwheat cages, while no effect was found in bees foraging on wild mustard and purple tansy (figure 2*d,e*). On day 2, FPF reduced flower visitation frequency independently of nutritional stress by 20% (figure 3), while no FPF effect was found on flight duration during this day. Similarly, FPF exposure decreased the proportion of females that immediately find their nest by 11% and 14% during days 1 and 2 independently of nutritional stress (figure 3). No main effect of FPF exposure or any synergistic effect with nutritional stress were found on day 9 (figure 3), while nutritional stress had an effect on various proxies of fitness during all 3 days (electronic supplementary material, table S1). Offspring survival (proportion of offspring that hatched in spring) was 51%, 79% and 86% for buckwheat, wild mustard and purple tansy cages, respectively (independently of the assessment day on which the brood cell was built). No main effect of FPF or any synergism with nutritional stress was found on offspring survival, sex ratio or body weight (electronic supplementary material, figures S2–S3 and table S2).

Approximately 12 h after FPF application, FPF residues in pollen-nectar provisions from purple tansy cages (lowest nutritional stress) were considerably higher with 41.7 ppm compared with the other two food plants: provisions from wild mustard and buckwheat cages had residue levels of 21.2 and 7.9 ppm, respectively.

## 4. Discussion

Overall, our results show that the novel insecticide FPF can have negative impacts on the survival, offspring production and foraging performance of solitary bees under field-realistic conditions when nutritional stress occurs. These effects may result in detrimental reductions of population sizes of solitary bee species [57–59] in environments where bees experience nutritional stress as e.g. in intensively managed agricultural landscapes with a low floral diversity and temporary lack of flowers. Resulting decreased bee abundances, together with the sublethal effects on foraging activity and flower visitation frequency, may lower pollination services to entomophilous crops [25,57]. Although negative effects of pesticides can be caused by co-formulants [60], the co-formulants in Sivanto Prime have not been found to be harmful to bees, suggesting that FPF is the driver of the adverse effects found. These effects probably resulted from high oral exposure after application as oral toxicity of FPF is relatively high compared to contact toxicity [35] and the percentage of foraging females during application was estimated below 10% compared to full bee flight. As furthermore no chronic effects were found nine days after application, restricting the application of FPF in bee-attractive crops to non-flowering stages could drastically reduce the risk of this insecticide for bees and other flower-visiting insects [61].

FPF is considered a potential successor of the neonicotinoids that have been banned in cropland of the European Union because of observed adverse impacts on bees and other non-target organisms [35]. Neonicotinoids can have manifold sublethal effects and impair the navigation memory, flight duration and foraging activity [5,6]. Here, we report for the first time similar negative impacts of the novel insecticide FPF on the solitary model bee species *O. bicornis*. Importantly, beyond such sublethal effects, FPF caused a mean mortality of 43% of adult female *O. bicornis* in food-stressed bees. Together, these effects could cause reductions of the total reproductive output above 40% when exposure occurs early during the reproductive season. Some neonicotinoids, in comparison, were found to cause decreases above 50% in the reproductive output of solitary bees even in the absence of food stress [8,57,58,62]. FPF therefore seems to be less harmful to solitary bees of the genus *Osmia* compared to the banned neonicotinoids. Nevertheless, we show that FPF, despite the short-lasting adverse effects, can drastically reduce population development when combined with food stress.

Moreover, our findings show that bees feeding on buckwheat flowers had the lowest capacity to clear FPF after exposure, followed by bees feeding on wild mustard and purple tansy, which probably contributed to the observed synergistic effects between nutritional stress and FPF exposure on various proxies of fitness of *O. bicornis*. Plant species can differ in various nutritional properties that can modulate the bees' response to pesticides. A low protein-lipid ratio and a high protein content of the diet can increase the tolerance towards pesticides [16,20]. Additionally, secondary metabolites, such as phenolic compounds or glucosinolates, can upregulate the detoxification and increase bees' resilience to pesticide exposure [19–21]. The higher pollen protein content of purple tansy and the lower protein-lipid ratio in combination with the presence of glucosinolates in wild mustard could therefore drive the increased resilience of bees towards FPF after foraging on these plant species. Compared to buckwheat, these two plant species also offered higher quantities of floral resources which may facilitate detoxification associated with an increased energetic investment [63]. Opposite to this hypothesis, a recent semi-field study with *Osmia lignaria* found only additive effects of neonicotinoid exposure and flower limitation [8]. However, a reduced resource availability per flower, in comparison to a reduced number of flowers, may strongly affect foraging efficiency and net energy intake. Thus, in our study, the reported synergistic effects between nutritional stress and FPF exposure may have been driven by a combination of resource availability and other nutritional properties of food plants.

FPF was classified as bee-safe as e.g. no negative effect of FPF on honeybee foraging activity or colony development were found when the insecticide was tested in a field study [64]. This risk assessment focused on honeybees as a model organism for pollinators and evaluated the effect of FPF in the absence of other stressors [26,27,29]. Here, by contrast, we found strong adverse effects of FPF on the solitary bee species *O. bicornis* under field-realistic conditions and show that effects depend on the food source provided. Nutritional stress substantially augmented the adverse impact of FPF on *O. bicornis* survival, reproductive success and foraging behaviour. These synergistic effects did not result from differences in FPF exposure between plant species as FPF caused the strongest adverse effects on *O. bicornis* when applied to buckwheat, which had the lowest residue levels in pollen-nectar provisions. In current risk assessments, however, higher tier (semi-)field studies do not consider potentially distinct impacts of pesticides applied to multiple crops [26,27]. Clearly, a higher tier (semi-)field study assessing the risk of FPF to *O. bicornis* would draw different conclusions depending on the food plant chosen. To reliably evaluate the risk of pesticides for bees, such synergistic effects need to be considered during pesticide risk assessments as bees in intensively managed agroecosystems dominated by crop monocultures are probably concomitantly exposed to pesticides and nutritional stress associated with available crops and a temporary lack of flowers [65].

By contrast, agricultural landscapes with high amounts of complementary floral resources can reduce pesticide exposure [13], offer a diverse and rich diet throughout the season that covers well the nutritional needs of pollinators and thereby, as indicated by our findings, also reduce bee susceptibility towards insecticides. This underpins the key role of conserving flower-rich semi-natural habitats and promoting agri-environment schemes to sustain populations of wild bee species in agroecosystems [66,67]. Our study strongly supports calls for a paradigm shift towards more holistic environmental risk assessment schemes for pollinators, that not solely focus on honeybees as a model species, and that account for interactive effects of agrochemicals with further anthropogenic stressors, such as food stress. Schemes ignoring the here demonstrated synergistic effects can fail to adequately protect bee pollinators and the vital pollination services they provide.

## Data Availability

Data available from the Dryad Digital Repository: https://doi.org/10.5061/dryad.stqjq2c69 [68]. The data are provided in electronic supplementary material [69].
